# Vertebral Erosion Secondary to Aortic Aneurysm

**DOI:** 10.1155/2020/6062140

**Published:** 2020-01-24

**Authors:** Zakaria Toufga, Fadma Aoujil, Nabil Moatassim Billah, Ittimad Nassar

**Affiliations:** Central Radiology Department, University Hospital Center Ibn Sina, Rabat, Morocco

## Abstract

We report the case of a patient who presented for back pain with paresthesia, and the CT showed vertebral lysis of aneurysmal origin. The aneurysm of the thoracic aorta compresses the anterior surface of the dorsal vertebrae and by mechanical effect is responsible for the destruction of the opposite bone. The knowledge of this cause is very important considering the frequency of other tumoral and infectious causes of this affection.

## 1. Introduction

In the majority of cases, pseudotumoral vertebral lysis is a radiological sign leading to a tumor origin (metastasis, myeloma) or infectious origin (tuberculosis). The aneurysmal origin is a cause that is potentially rarely described in the literature, chest CT angiography plays a key role in diagnosis, and the diagnostic range must include this etiology to prevent serious complications.

## 2. Case Report

We report a case of a 50-year-old patient with no medical or surgical history who has progressive worsening dorsalgia, with recent paresthesia of limbs and chest pain.

**Figure 1:**
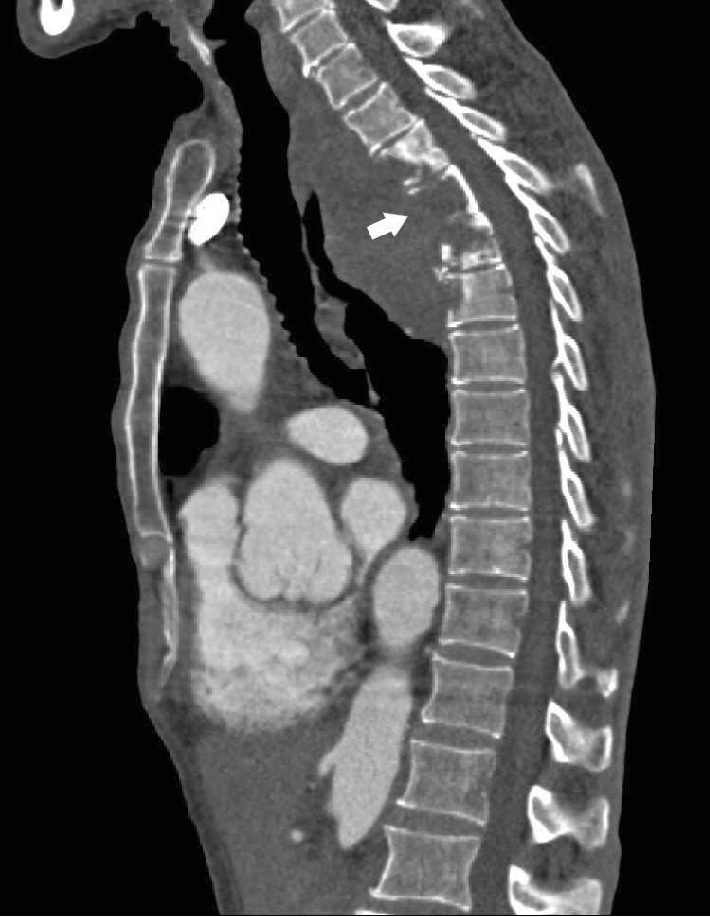
Sagittal section of a chest CT angiography showing the lysis of the vertebral bodies of D2, D3, D4, and D5 without displacement of the posterior wall.

**Figure 2:**
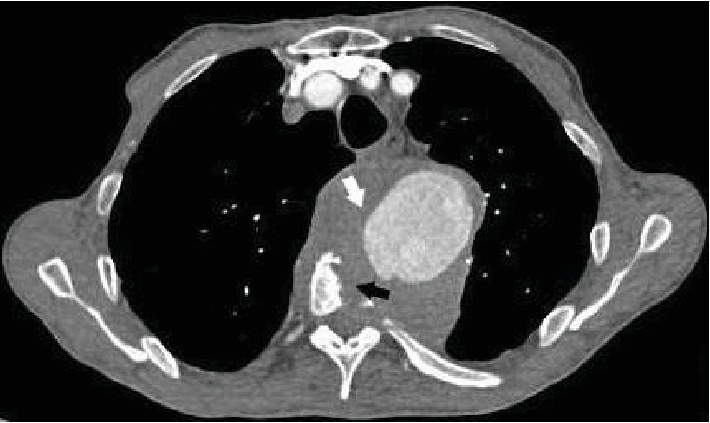
Axial section of a chest CT angiography showing aneurysmal formation (white arrow) implanted between the butt and the descending aorta, partially thrombosed coming into direct contact with the anterior surface of the vertebral bodies of the dorsal spine, responsible for bone lysis (black arrow) with a positive draped aortic sign.

**Figure 3:**
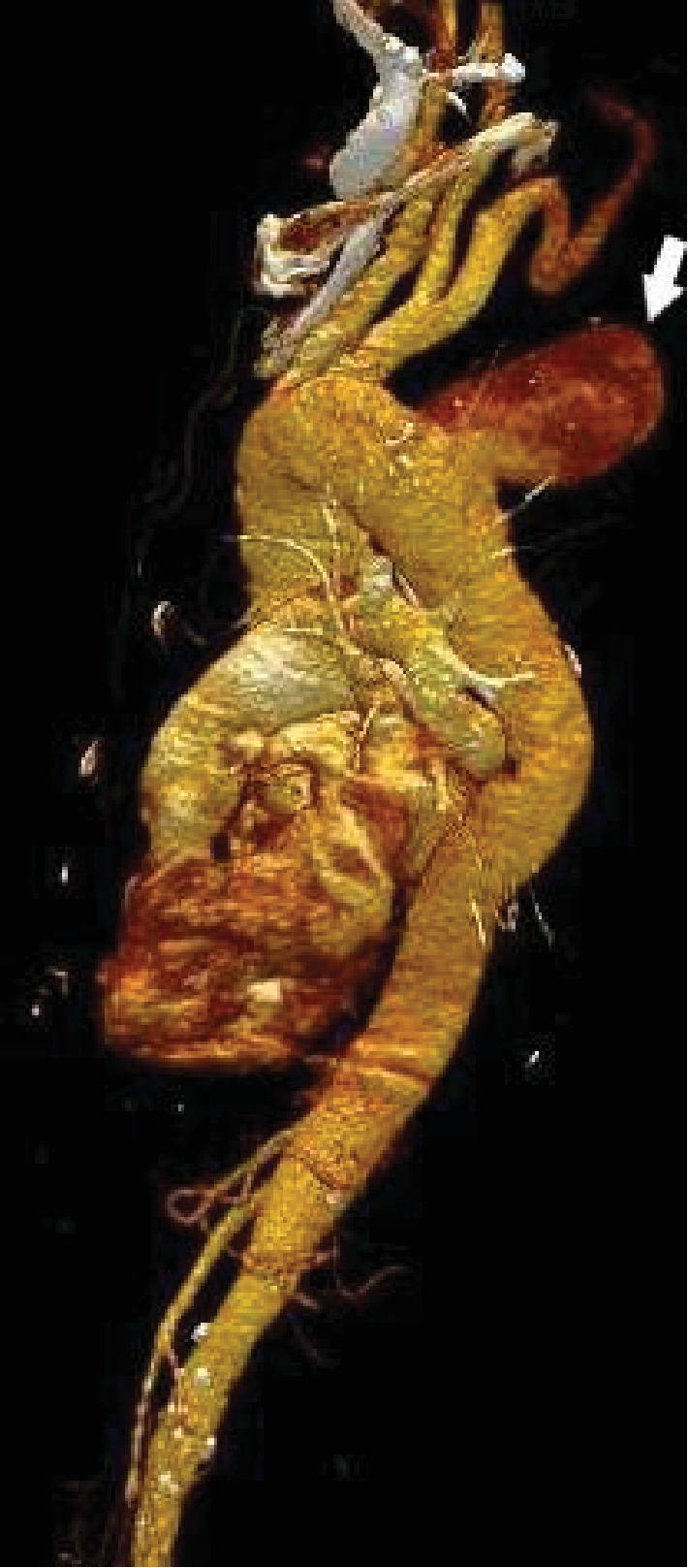
Reformation volume rendering technique showing the aortic aneurysm formation.

The clinical examination objectified back pain in the first dorsal vertebra with paresthesia of the limbs. The biological examination was normal. A radiograph of the dorsal and lumbar spine showed lysis of the vertebral bodies of D2, D3, D4, and D5. CT confirmed the lysis of the vertebral bodies of D2, D3, D4, and D5 (Figure 1), but it also objectified the presence of an aneurysmal sac implanted between the butt and the descending aorta, partially thrombosed, with direct contact with the destroyed face of the vertebral bodies of the dorsal spine (Figures 2 and 3).

## 3. Discussion

An aneurysm is a permanent and localized dilatation of an artery of more than 50% compared to the normal diameter, with a loss of parallelism of the edges [1]. According to the three segments of the thoracic aorta, the aneurysm can be found in 60% in the ascending aorta, 10% in the buttock, and 40% in the descending aorta [1].

Vertebral erosion secondary to an aortic aneurysm is most often located in the anterior region of the vertebral body; the suggested physiopathological mechanism is a repetitive mechanical pressure causing relative bone ischemia, leading to lysis and bone destruction [2]; the association with osteoporosis increases the risk of erosion. It occurs with a frequency of 7 to more than 25% of the cases and does not generally exceed 50% of the vertebral body [3].

The main signs that reveal the diagnosis are spinal pain and neurological deficits (paraparesis or paraplegia); however, infection and inflammation are not uncommon [4, 5].

The pseudotumoral aspect of bone erosion can orientate as in our case towards a tumoral (metastasis, multiple myeloma) or infectious (tuberculosis) origin [5].

The thoracoabdominal CT angiography is the reference examination, and it allows studying the whole thoracoabdominal aorta and its branches and assessing the relationship with the different adjacent structures (vertebrae, psoas, and the peritoneal retro); it also makes it possible to objectify the signs of aneurysmal rupture (intra- or retroperitoneal hematoma) or predictive signs of rupture, such as rupture of the continuity of parietal calcifications and the sign of the crescent or the sign of the draped aorta [6]; the latter is of great diagnostic value, and it is considered positive when the posterior wall of an aortic aneurysm is draped or molded on the anterior surface of the vertebra, with loss of the fat planes located between the aneurysm and the vertebra. Its presence indicates a weakening of the aortic wall and an imminent risk of rupture [5, 6].

Magnetic resonance imaging may be indicated in stable patients for comparative monitoring of lesions [1], but CT scans are more efficient for assessing bone lesions.

## 4. Conclusion

Spinal pain associated with vertebral osteolysis is most often directed towards tumor or infectious etiologies. The aneurysm of the aorta is a rare etiology but must be mentioned in order to avoid the evolution of serious complications.
